# Association of insertion /deletion polymorphism of ace gene with essential hypertension in patients of Khyber Pakhtunkhwa

**DOI:** 10.12669/pjms.40.3.7354

**Published:** 2024

**Authors:** Abdur Razaq, Ayesha Khan, Syed Tahir Shah, Sana Ullah

**Affiliations:** 1 Abdur Razaq, Institute of Pharmaceutical Sciences, Khyber Medical University, Peshawar, Pakistan; 2 Ayesha Khan, Department of Medicine, Combined, Military Hospital Nowshera, Nowshera, Pakistan; 3 Syed Tahir Shah, Cardiology Department, Kuwait Teaching Hospital, Peshawar, KPK, Pakistan; 4 Sana Ullah, Cardiology Department, Kuwait Teaching Hospital, Peshawar, KPK, Pakistan

**Keywords:** ACE gene, Insertion, Deletion, Hypertension

## Abstract

**Objective &Background:**

The exact cause of hypertension is unknown in about 90 to 95% patients, known as essential hypertension. Genes may play a crucial role in the pathology of essential hypertension. Gene for angiotensin converting enzyme (ACE) is found on long arm of chromosome 17q23, where 287 base pair insertion or deletion (I/D) polymorphism may occur. This study was aimed to assess the association of I/D polymorphism of ACE gene with blood pressure (BP) in Patients of Khyber Pakhtunkhwa (KPK).

**Methods:**

This Descriptive Cross-sectional study was conducted from 1^st^ June 2021 to 30^th^ September 2021 at Kuwait Teaching Hospital, Peshawar. The genomic DNA was extracted from lymphocytes and PCR was performed for identification of ACE I/D polymorphism.

**Results:**

Total 181 individuals (121 Hypertensive and 60 normal) were enrolled in the study. The measured systolic and diastolic BP in cases were 153.91mmHg±12.65 and 92.94mmHg±5.72, respectively while in control were 118.20±17.13 and 74.12mmHg±7.58, respectively. The Deletion Homozygous (DD), Insertion Homozygous (II) and Deletion and Insertion Heterozygous (DI) genotypes in hypertensive patients were 47 (38.84%), 17 (14.04%) and 57 (47.10%) respectively while in Control group the DD, II and DI were 4 (6.66%), 25 (41.66%) and 31 (51.66%) respectively. This study showed association of DD genotypes of the ACE gene with hypertension as compared to healthy individuals.

**Conclusion:**

Individuals with DD genotype may have association with hypertension. polymorphism of ACE gene was proved to be an important genetic marker for essential hypertension in Patients of KPK.

## INTRODUCTION

Essential Hypertension is one of the major risk factors for the cardiovascular disease morbidity and mortality.^1^ Hypertension can be defined as when the systolic blood pressure (SBP) of a person in the office or clinic is 140mmHg and the diastolic blood pressure is more than 90mmHg.^2^ Worldwide 1.28 billion adults are hypertensive of low- and middle-income countries and approximately 46% adults are unaware of their diagnosis.^3^ Hypertension is a major global chronic non-communicable disease. One-quarter of the world’s adult population has hypertension, and this is likely to increase to 29% by 2025.

The absolute prevalence of hypertension in economically developed nations is 37.3% compared with 22.9% in developing nations.^4^ Prevalence of hypertension in Pakistani urban and rural areas is 44.3% and 46.8%, respectively.^5^ Several life style factors have association with essential hypertension like obesity, diet. About 50 to 60% of patients are sensitive to salt due to genetics and there is chance to develop essential hypertension.^6^ It is estimated that 30-50% of hypertension is due to genetics and up to now only 2-3% of polymorphism has been found.^7^ Among various genes polymorphisms ACE gene have association with hypertension.^8^ Angiotensinogen is the precursor of angiotensin and its production occur in the liver.

Its conversion occurs to angiotensin I by renin. It is a weak vasoconstrictor and it convert to Angiotensin-II which is a potent vasoconstrictor. Angiotensin-II physiologic responses include decreased endothelial nitric oxide (NO) activity and increased norepinephrine release both leads to peripheral and renal vasoconstriction. Angiotensin-II acts on adrenal cortex and promote the release of aldosterone. Aldosterone activates sodium-potassium ATPase pump in nephrons in the result of which potassium depletion and sodium retention occur resulting systemic volume expansion and hence blood pressure increases. The Proposed mechanisms of angiotensin-II are of two types: hemodynamic and non-hemodynamic effects.

Hemodynamic effects are systemic hypertension, systemic and renal vasoconstriction, mesangial cell contraction and increase pressure in glomerular capillary. Non-hemodynamic effects are renal hypertrophy and cell proliferation, stimulation of cytokines (e.g., VEGF, endothelin) and stimulation of superoxide production.^9^ Gene responsible for ACE in human is found on long arm of chromosome 17q23, and there is 287 base pair insertion or deletion (I/D) polymorphism (rs179975) in intron 16. The ACE gene is 21 kilo bases (kb) long and consists of 25 introns and 26 exons.^10^

There are various contradictory results of I/D polymorphism of the ACE gene with HTN that may be due to ethnic differences and gender.^11^ However, to date the I/D polymorphism of ACE gene in Khyber Pakhtunkhwa (KPK), Pakistani subjects has not been studied. This study will help us in selecting proper antihypertensive drug for patients of Hypertension in KPK. Therefore, this study was designed to find the association of I/D polymorphism of the ACE gene in essential hypertensive patients in KPK Pakistani subjects.

## METHODS

This Descriptive Cross-sectional study includes a total of 181 individuals (121 cases Hypertensive and 60 control normal blood pressures). Prevalence of hypertension in Pakistan is 29.22% with sample size of 219.^12^, so assuming sample size on that bases 181 Patients of KPK having age 50-70 years presenting to Outpatient department (OPD) were included. Patients were labeled Hypertensive if they were using antihypertensive drugs for last six months. Patients having past history of kidney disease were excluded from the study. A detailed Performa for demographic and clinical data was prepared along with consent form.

The Performa contains information like name, age, gender, ethnicity, duration of disease, contact number as well clinical record of the patients. The demographic and clinical data were obtained at the time of measuring blood pressure, weight and height. The following physiological and clinical variables were measured; Weight, Height, BMI (weight in Kg/height in m^2^), Systolic blood pressure and diastolic blood pressure were measured with the help of Mercury Sphygmomanometer three times of all patients in sitting position. 3 to 5cc blood were taken by venipuncture and was transferred to ethylenediaminetetraacetic acid (EDTA) tubes and kept at -20ºC for further study.

This study was conducted from 1^st^ June 2021 to 30^th^ September 2021 at Cardiology Department, Kuwait Teaching Hospital, Peshawar Pakistan and further experimental work was done in Institute of Basic Medical Sciences (IBMS), Khyber Medical University (KMU), and Peshawar Pakistan.

### Ethical Approval:

Ethical committee (EC) of KMU, vide letter no. DIR/KMU-EB/A1/000429 has approved this study. Informed consent was taken from all patients.

### Determination of ACE I/D Polymorphism:

The genomic DNA was extracted from peripheral blood lymphocytes using salting out techniques.^13^ For identification of ACE I/D polymorphism polymerase chain reaction (PCR) was performed of all samples. Extracted DNA amplification was carried out in 20ul volume. The reaction mixture contains master mix 10ul, deionized water 8.9ul, primer forward 0.3ul, primer reverse 0.3ul and DNA template 0.5ul. The forward primer was 5’-CTGGAGAGCCACTCCCATCCTTTCT-3’ and reverse primer was 5’- GACGTG GCCATCACATTCGTCAGAT-3’ were used. PCR cycling conditions were initial denaturation at 94c for five minutes one cycle, followed by 35 cycles at 94c for one minute (melting).

**Fig.1:**
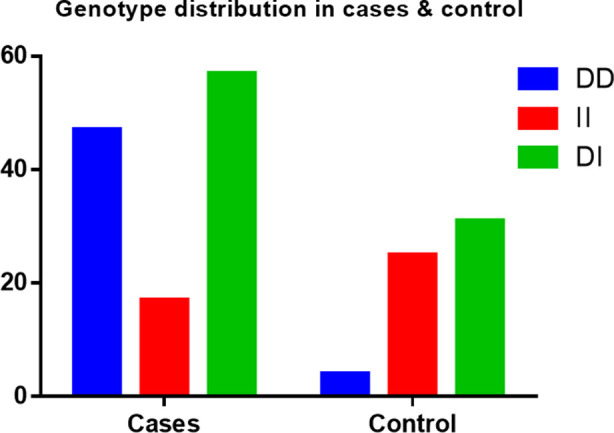
ACE genotype distribution aong cases and controls.

Conditions for annealing were 58c for 45 seconds, extension at 72c for one minute and final extension at 72c for eight minutes one cycle. The amplified products were analyzed on 2% agarose gel containing 5ul ethidium bromide. Gel was visualized under UV spectrophotometer. Three different types of genotypes bands were identified on gel. Bands at 190bp were DD homozygous, at 490bp II homozygous and at 190bp and 490bp both were D/I heterozygous (Fig.1). Twelve samples were randomly taken for sequencing. Finch TV software was used for analysis There were no peaks in all chromatograms of ‘N’ basecaller, so no SNP found in any sample.

### Statistical Analysis:

Statistical Package for Social Sciences (SPSS) version 20.0 and Microsoft Excel 2013 were used to analyze the means and standard deviations of cases and control. Demographics data of both groups as well as their percentages were measured. Independent sample T-test was applied to determine the difference between demographics of patients and controls. Fisher’s exact test was done to find any possible association between genotypes of ACE gene and hypertension. Graph-pad prism version six was used to construct graphs. All the test values were evaluated by two-tailed method. P value less than 0.05 was considered to be significant.

## RESULTS

One hundred eighty one cases include 55% females and 45% males while in control group 40% and 60% were females and males respectively. The demographic data of patients and controls are summarized along with their statistical difference in Table-I.

**Table-I:** Various characteristics of Hypertensive patients and Control.

Variable	Disease state	Mean + SD	95% CI	p-value
Gender (M/F)	Yes (121) 54/67	-	-	-
	No (60) 36/24			
Age (Years)	Yes (121)	58 ± 7.2	0.51 to 4.64	0.04
	No (60)	55.4 ± 5.1		
Weight (Kg)	Yes (121)	69.9 ± 8.2	-10.74 to -3.95	<0.001
	No (60)	77.3 ± 14.7		
Height (cm)	Yes (121)	169.4 ± 7.3	0.44 to 5.36	0.02
	No (60)	166.5 ± 8.8		
BMI	Yes (121)	24.3 ± 3.1	-4.99 to -2.5	<0.001
	No (60)	28.1 ± 5.2		

Legend HTN, Hypertension; BMI, Body Mass Index.

The biochemical analysis of cases and control are summarized in Table-II. The mean, standard deviation and range of Systolic blood pressure (SBP) and diastolic blood pressure (DBP) of Hypertensive patients and control were 153.91mmHg±12.65, 50.00, 92.94mmHg± 5.72, 30, 118.20mmHg±17.13, 100.00, 74.12mmHg±7.58, 40.00 respectively as explained in Table-II.

**Table-II:** Biochemical analyses of Cases and Control.

Investigation	Cases	Control
SBP (mmHg)		
Mean ± SD	153.91±12.65	118.20±17.13
Range	50.00	100.00
DBP (mmHg)		
Mean ± SD	92.94±5.72	74.12±7.58
Range	30	40.00

Legend: SBP: Systolic blood pressure; DBP: Diastolic blood pressure.

In Hypertensive patients the DD, II and DI genotypes were 47 (38.84%), 17 (14.04%) and 57 (47.10%) respectively. In Control group the DD, II and DI were four (6.66%), 25 (41.66%) and 31 (51.66%) respectively as explained in Table-III and shown in Fig-1.

**Table-III:** Genotypes distribution among Cases and Control.

Groups	DD – n(%)	II – n(%)	DI – n(%)
HTN (N=121)	47 (38.84)	17 (14.04)	57(47.10%)
Control (N= 60)	4 (6.66)	25 (41.66)	31(51.66%)

Legend HTN, Hypertensive; DD, deletion homozygous; II, insertion homozygous; DI, deletion insertion heterozygous.

**Fig.2:**
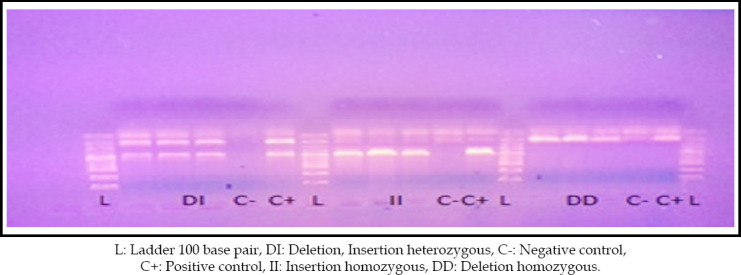
ACE gene polymorphisms.

In order to find out any possible statistic association between cases and controls based on ACE gene polymorphism, fisher’s exact test was applied. The results are shown in Table-IV. Fig.2 shows the gel electrophoresis image showing different ACE genotypes.

**Table-IV:** Association between ACE genotypes in cases and control.

Association	Genotypes	Cases	Control	p-value	OR (95%CI)
DD vs II	DD	47	04	<0.001	17.2(5.23-56.95)
	II	17	25		
DD vs DI	DD	47	04	<0.001	6.3(2.1-19.4)
	DI	57	31		

## DISCUSSION

Essential HTN is a multifactorial disorder caused by genetic, demographic and environmental factors.^14^ Renin Angiotensin Aldosterone System (RAAS) gene polymorphisms have been studied in different ethnic groups to find the genetic susceptibility to HTN.^15^ There is 287 bp insertion or deletion of ACE gene in the 16^th^ Intron of chromosome number 17 which result increased plasma and serum ACE level. The insertion/ presence of 287 bp is represented as homozygous-II, ID for heterozygous, while DD represents the deletion/ absence of a 287 bp. There is strong association of Insertion/deletion(I/D) polymorphism in ACE gene with essential hypertension.^16^

DD genotypes and D allele as compared to II genotype and I allele have strong association with HTN.^17^ Individuals with homozygous (DD genotype) have the highest amount of ACE in serum, those homozygous insertion (II genotype) have the lowest amount of serum ACE level, while heterozygous (ID genotype) have intermediate level.^18^ Punjabi population of Faisalabad region with DD genotype are proven to hypertension while those who are ID genotype have association with systolic blood pressure.^19^ Another study on population of district Kohat only, Khyber Pakhtunkhwa showed no association of ACE gene polymorphism with hypertension.^20^

The DD genotype in our population in 121 patients was 47 (38.84%) while II genotype was 17 (14.04%). In control out of 60 only four (6.66%) were DD and 25 (41.66%) were II genotype while other Pakistani study showed that DD genotype of ACE gene was 28.8%.^21^ Study on Punjabi Population of Pakistan showed that ACE ID polymorphism have association with hypertension (OR: 2.844: CI: 1.32–6.110). 22

A similar study was done in Islamabad tertiary, Pakistan that observed no overall significant differences between the I/D, I/I, and D/D genotypes of ACE with P = 0.413). The frequency of the ACE I/I (homozygous) genotype was significantly higher in hypertensive patients in comparison of control P = 0.041).^23^ North Indian population with DD genotype are more proven to essential hypertension.^24^

Another similar study was done in south Indian Population where the distribution of II, ID, DD genotypes of ACE gene was 28.3%, 32.6% and 38.9% respectively in essential hypertensive patients and 53.6%, 26.3% and 20% in controls.^25^

Study in Poland showed that ACE insertion/deletion polymorphism have no association with hypertension.^26^ The clinical characteristics of the essential hypertensive patients and control as well the distribution of ACE genotypes in relation to age, weight, BMI, SBP and DBP with respect to angiotensinogen gene polymorphisms are studied in Malaysian hypertensive subjects^27^ Taiwanese^28^ and Turkish populations.^29^

In our study, we found that DD genotypes of the ACE gene were strongly associated with HTN as compared to healthy individuals (p <0.001).Similar study was done on Chines population with (p <0.05).^30^ Due to the above controversial results, we planned our study to find the association of ACE gene polymorphism with HTN in KPK Pakistani population which has not been studied yet. The demographic data showed that there was significant difference in weight of control and cases with P-value **<**0.001. The difference in height and BMI of cases and control was also significant with P-value 0.02 and **<**0.001 respectively as explained in Table-I. The negative associations and discrepancies may be due to environmental factors, the racial differences or heterogeneity of the population and sampling biasness.^31^

Our study results suggest that more than one third of patients (38.8%) have higher level of serum ACE level due to their Deletion Homozygous (DD) genetics composition in KPK population which make ACE inhibitors as a first line antihypertensive medication in such patients.

### Limitations:

The study sample size was relatively small as compared to other epidemiological and association studies. It was not in randomized control trial like no matched control of age and sex were used. However, our study supports the Hypothesis that DD genotype of ACE gene has strong association with HTN. Further studies with larger sample size will confirm the association of I/D polymorphism of ACE gene with essential HTN in KPK Pakistani subjects.

## CONCLUSION

This study provides strong evidence for the association of I/D polymorphism of ACE gene in KPK Pakistani population with essential hypertension. The DD genotype has strong association with HTN and the D allele of the I/D polymorphism of ACE gene is proved to be an important genetic marker for essential HTN in KPK Pakistani subjects.

### Author`s Contribution:

**AR & SU:** conceived the experiment.

**AR, SU & AK:** were involved in data collection, statistical analysis and drafted the manuscript.

**AR, STS & SU:** reviewed the manuscript

**STS & AR:** responsible for the accuracy and integrity of the work.

## References

[1] Zhang Q, Cong M, Wang N, Li X, Zhang H, Zhang K (2018). Association of angiotensin-converting enzyme 2 gene polymorphism and enzymatic activity with essential hypertension in different gender:a case–control study. Medicine.

[2] Unger T, Borghi C, Charchar F, Khan NA, Poulter NR, Prabhakaran D (2020). 2020 International Society of Hypertension global hypertension practice guidelines. Hypertension.

[3] Pokharel Y, Karmacharya BM, Neupane D (2022). Hypertension—A Silent Killer Without Global Bounds:What Next?. J Am Coll Cardiol.

[4] Kearney PM, Whelton M, Reynolds K, Muntner P, Whelton PK, He J (2005). Global burden of hypertension:analysis of worldwide data. Lancet.

[5] Basit A, Tanveer S, Fawwad A, Naeem N, Members N (2020). Prevalence and contributing risk factors for hypertension in urban and rural areas of Pakistan;a study from second National Diabetes Survey of Pakistan (NDSP) 2016–2017. Clin Exp Hypertens.

[6] Iqbal AM, Jamal SF (2022). Essential hypertension. StatPearls [Internet]:StatPearls Publishing.

[7] Russo A, Di Gaetano C, Cugliari G, Matullo G (2018). Advances in the genetics of hypertension:the effect of rare variants. Int J Mol Sci.

[8] Ejaz S, Ali A, Riffat S, Mahmood A, Azim K (2021). Genetic polymorphism of the prostasin gene in hypertensive pregnant Pakistani females. Pak J Med Sci.

[9] Leehey DJ, Singh AK, Alavi N, Singh R (2000). Role of angiotensin II in diabetic nephropathy. Kidney Int.

[10] Pinheiro DS, Santos RS, Jardim PCV, Silva EG, Reis AA, Pedrino GR (2019). The combination of ACE I/D and ACE2 G8790A polymorphisms revels susceptibility to hypertension:A genetic association study in Brazilian patients. PloS One.

[11] Ahmad Yusof H, Che Muhamed AM (2021). Angiotensin-converting enzyme (ACE) insertion/deletion gene polymorphism across ethnicity:a narrative review of performance gene. Sport Sci Health.

[12] Ishtiaq S, Ilyas U, Naz S, Altaf R, Afzaal H, Muhammad SA (2017). Assessment of the risk factors of hypertension among adult &elderly group in twin cities of Pakistan. J Pak Med Assoc.

[13] Madhad VJ, Sentheil K (2014). The Rapid &Non-Enzymatic isolation of DNA from the Human peripheral whole blood suitable for Genotyping. Eur J Biotechnol Biosci.

[14] Lifton RP, Gharavi AG, Geller DS (2001). Molecular mechanisms of human hypertension. Cell.

[15] Mengesha HG, Petrucka P, Spence C, Tafesse TB (2019). Effects of angiotensin converting enzyme gene polymorphism on hypertension in Africa:A meta-analysis and systematic review. PloS One.

[16] Birhan TA, Molla MD, Abdulkadir M, Tesfa KH (2022). Association of angiotensin-converting enzyme gene insertion/deletion polymorphisms with risk of hypertension among the Ethiopian population. Plos One.

[17] Hristova M, Stanilova S, Miteva L (2019). Serum concentration of renin-angiotensin system components in association with ACE I/D polymorphism among hypertensive subjects in response to ACE inhibitor therapy. Clin Exp Hypertens.

[18] Lu M, Zhang J, Li M, Ge X, Dai X, Zhao J (2016). The angiotensin-I converting enzyme gene I/D variation contributes to end-stage renal disease risk in Chinese patients with type 2 diabetes receiving hemodialysis. Mol Cell Biochem.

[19] Hussain M, Awan FR, Gujjar A, Hafeez S, Islam MJC, Hypertension E (2018). A case control association study of ACE gene polymorphism (I/D) with hypertension in Punjabi population from Faisalabad, Pakistan. Clin Exp Hypertens.

[20] Saeed K, Muhammad N, Haris M, Hussain S, Syeda I, Hussain S (2019). ACE Gene Polymorphism in Hypertension Patients from District Kohat.

[21] Javaid A, Mansoor Q, Bilal N, Bilal A, Shaukat U, Ismail M (2011). ACE gene DD genotype association with obesity in Pakistani population. Bioautomation.

[22] Nawaz SK, Hasnain S (2011). Effect of ACE polymorphisms on the association between noise and hypertension in a Pakistani population. Journal of the Renin-Angiotensin-Aldosterone System.

[23] Ismail M, Akhtar N, Nasir M, Firasat S, Ayub Q, Khaliq S (2004). Association between the angiotensin-converting enzyme gene insertion/deletion polymorphism and essential hypertension in young Pakistani patients. BMB Rep.

[24] Abbas S, Raza ST, Chandra A, Rizvi S, Ahmed F, Eba A (2015). Association of ACE, FABP2 and GST genes polymorphism with essential hypertension risk among a North Indian population. Ann Human Biol.

[25] Krishnan R, Sekar D, Subramanium S (2016). Association of angiotensin converting enzyme gene insertion/deletion polymorphism with essential hypertension in south Indian population. Genes Dis.

[26] Pachocka L, Wlodarczyk M, Klosiewicz-Latoszek L, Stolarska I (2020). The association between the insertion/deletion polymorphism of the angiotensin converting enzyme gene and hypertension, as well as environmental, biochemical and anthropometric factors. Rocz Państw Zaki Hig.

[27] Ramachandran V, Ismail P, Stanslas J, Shamsudin N, Moin S, Mohd Jas R (2008). Association of insertion/deletion polymorphism of angiotensin-converting enzyme gene with essential hypertension and type 2 diabetes mellitus in Malaysian subjects. J Renin Angiotensin Aldosterone System.

[28] Tseng C-H, Tseng C-P, Chong C-K, Sheu J, Cheng J (2007). Angiotensin-converting enzyme gene polymorphism and stroke in type 2 diabetic patients in Taiwan. Europ J Clin Investig.

[29] Simsek S, Tekes S, Turkyilmaz A, Tuzcu A, Kılıc F, Culcu N (2013). Angiotensin-converting enzyme gene insertion/deletion polymorphism with metabolic syndrome in Turkish patients. J Endocrinol Investig.

[30] Li Y (2012). Angiotensin-converting enzyme gene insertion/deletion polymorphism and essential hypertension in the Chinese population:a meta-analysis including 21 058 participants. Int Med J.

[31] Persu A (2006). Candidate gene studies:accepting negative results. J Hypertens.

